# SDS-PAGE-Based Quantitative Assay for Screening of Kidney Stone Disease

**DOI:** 10.1007/s12575-009-9007-y

**Published:** 2009-05-15

**Authors:** Lau Wai-Hoe, Leong Wing-Seng, Zhari Ismail, Gam Lay-Harn

**Affiliations:** 1 School of Pharmaceutical Sciences, Universiti Sains Malaysia, USM, 11800, Penang, Malaysia; 2 Lam Wah Ee Hospital, Penang, Malaysia

**Keywords:** Kidney stone disease, SDS-PAGE, Tamm Horsfall Protein and diagnostic device

## Abstract

Kidney stone disease is a common health problem in industrialised nations. We developed a SDS-PAGE-based method to quantify Tamm Horsfall glycoprotein (THP) for screening of kidney stone disease. Urinary proteins were extracted by using ammonium sulphate precipitation at 0.27 g salt/mL urine. The resulted pellet was dissolved in TSE buffer. Ten microliters of the urinary proteins extract was loaded and separated on 10% SDS-PAGE under reducing condition. THP migrated as single band in SDS-PAGE. The assay reproducibility and repeatability were 4.8% CV and 2.6% CV, respectively. A total of 117 healthy subjects and 58 stone patients were tested using this assay, and a distinct cut-off (*P* < 0.05) at 5.6 μg/mL THP concentration was used to distinguish stone patients from healthy subjects. The sensitivity and specificity of the method were 92.3% and 83.3%, respectively.

## 1. Introduction

Pathogenesis of kidney stone disease involves nucleation, growth, aggregation, and retention of crystals in the kidney. Biochemical tests for urinary electrolytes cannot specifically identify the formation of stone in the kidney. In human, the mechanism against the formation of kidney stone involves macromolecules, i.e., protein and glycosaminoglycan that are polyanionic molecules with substantial amounts of acidic amino acid residues that inhibit crystal aggregation [1]. Tamm Horsfall glycoprotein (THP) is one of the inhibitors to crystalize aggregation in kidney [2,3].

Tamm Horsfall glycoprotein is the most abundant urinary protein in healthy individual [4,5]. It is excreted by the thick ascending limb of the loop of Henle [6]. THP is excreted in urine at a rate of ~50 mg/day and can be influenced by many factors including urine volume, diet, and exercise [7]. THP has a molecular weight of ~85 kDa although inclines to form macroaggregates of several million Daltons [8]. THP is heavily glycosylated by *N*-linked glycans that account for about 30% of its molecular weight [9]. Kidney stone patients excrete THP molecules lacking terminally linked sialic acid that reduces its inhibitory activity against stone formation [10]. Although reduced urinary THP excretion in stone patients had been documented [11-13], quantitative analysis of THP by using electroimmunodiffusion shown no changes in THP excretion levels between stone patients and healthy subjects [14,15]. Other studies by using radioimmunoassay and ELISA shown that THP excretion was inconsistent or reduced in stone patients [17-19] compared to the healthy subjects [16]. In the studies where analysis of THP was carried out by using SDS-PAGE, Yokomizoi *et al. *[11] showed that THP intensity in stone patients was lower than that of healthy subjects while Pourmand et al. [20] stated there was no significant difference in THP excretion between the two cohorts. In our earlier study, we had reported that THP can be used to differentiate kidney stone patients from healthy subjects [21]. The controdictory data obtained by these various studies may be due to the different proportion of free and aggregated forms of THP in patients' urine; furthermore, the two THP forms have different solubility in salts solutions [7].

The SDS-PAGE-based method for quantification of protein is a method of choice due to its simplicity and reliability, whereby proteins are resolved and migrated according to their molecular weights [12]. It has been widely used for analysis of THP in kidney stone disease [11,12,20]. In this present study, we aim to develop a non-invasive SDS-PAGE quantitative method that can be used reliably for screening of kidney stone disease; THP will be used as the quantifying marker. THP aggregation will be overcome by the use of a suitable buffer. Finally, the usability of the developed method as a screening assay for kidney stone disease will be validated and evaluated by using urine from healthy subjects and stone patients.

## 2. Methods

### 2.1. Urine Specimen Collection

Midstream urine samples were collected from 58 stone formers (34 male, 24 female), age range 28–74 years and 117 healthy subjects (41 male, 76 female) age range 20–86 years. The urine samples were provided by the Urology Clinic, General Hospital, Pulau Pinang, and the Lam Wah Ee Hospital, Pulau Pinang, Malaysia. All stone patients had been confirmed by radiography using plain abdominal radiography (KUB) and/or intravenous urography (IVU). Stone patients were afflicted by partial obstruction and non obstruction kidney stones. Control subjects were asymptomatic of kidney stone and had no previous history of kidney stone or other renal related diseases, their urine FEME (microscopic) test showed no red blood cells present and included staff and students in the School of Pharmaceutical Sciences, USM, and nurses from the nursing institute of Lam Wah Ee Hospital, Pulau Pinang. Exclusion criteria for this study are urine samples with urinary creatinine concentration <2.00 mmol/L and >19.00 mmol/L. Normal range is 2.00–19.00 mmol/L based on the Cayman Chemical Creatinine Assay. Urine sample was aliquoted in 1 mL/microcentrifuge tubes and stored at -20°C before analysis. All the urine samples were analyzed for THP concentration using ELISA [21].

### 2.2. Urinary Creatinine Assay

Creatinine concentration in urine sample was measured by Cayman Chemical Creatinine Assay (Cayman Chemical Company) with slight modification. The assay was relied on the Jaffe' reaction, wherein a yellow or orange color was formed when the metabolite was treated with alkaline picrate [22]. The color derived from creatinine was then destroyed at acidic pH. The difference in color intensity was measured at 495 nm before and after acidification is proportional to the creatinine concentration [23]. A creatinine standard curve was constructed for determination of creatinine concentration in urine samples.

### 2.3. Urinary protein extraction

Urinary proteins were extracted from 1 mL urine samples by ammonium sulphate salt precipitation [24]. The aliquot urine (1 mL) was thawed to room temperature and added with 0.27 g ammonium sulphate. After gentle vortex for 2 min, the urine was centrifuged (10,000 *g*, 20 min at 20°C) and the pellet was then collected. The pellet was dissolved in 80 μL TSE buffer [10 mM Tris, 1 mM EDTA, 1% (w/v) SDS, pH 8.8] and 20 μL reducing sample buffer [0.5 M Tris-HCl at pH 6.8, 20% (v/v) glycerol, 10% (w/v) SDS, 0.5% (w/v) bromophenol blue and 5% β-mercaptoethanol]. Urinary protein was solubilized and reduced by heating at 100°C for 10 min before loading unto gels.

### 2.4. Standard THP Preparation and Standardization

The Standard THP sample was prepared from the dilution of commercial THP (Biomedical Technologies; 1 μg/μL) to 0.5 μg/mL. The QC was prepared from pooled normal urine by ammonium sulphate salt precipitation. The QC was used in method validation for precision of the assay. The pellet was dissolved in TSE buffer, solubilized and reduced in reducing sample buffer with 5% β-mercaptoethanol. The concentration of QC is 0.29 μg/mL as calculated relative to the standard based on band intensity. Ten microliters of the standard THP and the samples were run concurrently in a similar gel. The relative band intensity of the standard THP to THP from the sample was used to calculate the concentration of urinary THP in each sample.

### 2.5. SDS-PAGE for Urinary THP Measurement

Urinary proteins were resolved by SDS-PAGE according to a modified Laemmli method [25]. Electrophoresis was performed in vertical mini-slab gel (Mini-Protean III; BioRad) with a gel thickness of 0.75 mm and gel size 8 × 7 cm. The gels were composed of 10% resolving gel and 4% stacking gel and run at constant voltage of 200 volts for 50 min.

Amounts of 10 μL of protein extracts were loaded into each well of the gel. The same volume of standard THP was added in a separate well alongside the samples. After electrophoretic separation, the gel was stained with Coomassie blue solution (0.01% Coomassie brilliant blue R250, 45% (v/v) methanol and 10% (v/v) glacial acetic acid) for 30 min at room temperature and subsequently destained in the destaining solution (50% (v/v) methanol and 2% (v/v) acetic acid) for 1 h. The gel image was captured and analysed using VersaDoc Imaging System (BioRad). THP band intensity relative to standard THP was used to calculate THP concentration in sample by using Quantity One 1-D software (BioRad).

### 2.6. Assay Validation

The developed SDS-PAGE was validated for assay precisions, i.e., repeatability and reproducibility [26]. The repeatability (within day precision) was calculated by the coefficient of variation (CV) for QC THP that was run on the same day, whereas reproducibility (between day precision) was calculated from the average of the within assay precision of QC THP analyzed in three runs at different day. The cut-off points to differentiate stone formers and healthy subjects was determined by using trained urine from 58 stone formers and 117 healthy subjects, where the THP concentrations of these urine samples were previously measured using the ELISA method [21]. We defined true positive (TP) as the stone formers' urine (from patient' clinical data) with THP concentration below the cut-off value set, while true negative (TN) was set as the healthy subjects' urine with THP concentration above the cut-off value. In contrast, false positives (FP) were healthy subjects' urine with THP concentration below the cut-off value and vice-versa with false negative (FN) urine. Sensitivity is calculated as TP/(TP + FN) whereas specificity is TN/(TN + FP) [26]; the results of both calculations are expressed as percentages.

### 2.7. Data Analysis

Data analysis was performed using SPSS v. 15.0. For comparison between cohorts, Student's t test and Chi-square test were employed and the chosen level of statistical significant was at *P* = 0.05. All the value were presented as mean ± standard error of the mean (SE).

## 3. Result

**Figure 1:**
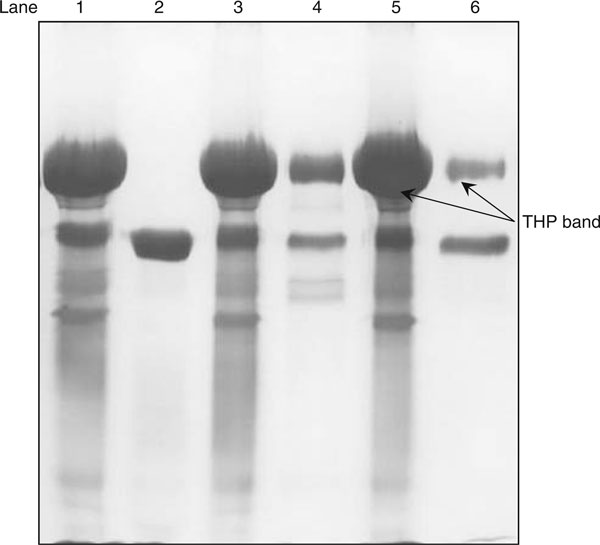
**Amount of ammonium sulphate salt [(NH_4_)_2_SO_4_] required for urinary proteins recovery**. Lane 1: pellet fraction of 0.27 g (NH_4_)_2_SO_4_; lane 2: supernatant fraction of 0.27 g (NH_4_)_2_SO_4_; lane 3: pellet fraction of 0.13 g (NH_4_)_2_SO_4_; lane 4: supernatant fraction of 0.13 g (NH_4_)_2_SO_4_; lane 5: pellet fraction of 0.07 g (NH_4_)_2_SO_4_; and lane 6: supernatant fraction of 0.07 g (NH_4_)_2_SO_4_. The gel was silver stained.

**Figure 2:**
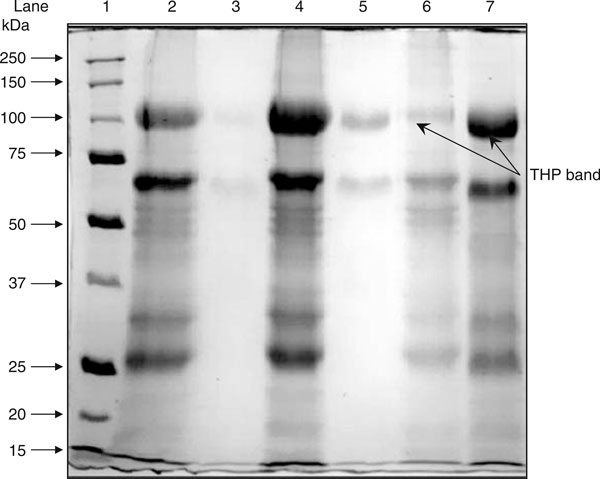
**Solubility of salt precipitated urinary proteins in distilled water, 0.01 M PBS and TSE buffer**. Lane 1: molecular weight markers; lane 2: pellet fraction of distilled water buffer; lane 3: supernatant fraction of distilled water buffer; lane 4: pellet fraction of 0.01 M PBS buffer; lane 5: supernatant fractions of 0.01 M PBS buffer; lane 6: pellet fractions of TSE buffer; and lane 7: supernatant fractions of TSE buffer. Salt-precipitated urinary proteins were dissolved in the indicated buffers. After centrifugation, the recovered proteins in the pellet fractions and supernatant fractions were separated by using SDS-PAGE. The gel was stained with Coomassie blue.

Sample preparation is an important part of the development of a quantatitive method. In this study, THP in urine was recovered by the salt-precipitating technique. Complete recovery of THP in 1 mL urine of healthy subjects was achieved by salting out using 0.27 g of ammonium sulphate (Figure 1). At this salt to urine ratio, an intense THP band was detected in the pellet and faint THP band, if detectable in the supernatant fraction, where >99% of urinary THP was recovered (Table 1). Figure 2

**Table 1:** Percentage of urinary THP recovered after precipitated with ammonium sulphate

	**Intensity (intensity × mm**^**2**^**)**		
		
**Amount of (NH**_**4**_**)**_**2**_**SO**_**4 **_**added for precipitation (g)**	Pellet fractions (P)	Supernatant fractions (S)	Percentage of THP recovered 100% - [S/(P+S) × 100%]
0.27	13,883.81	24.77	99.8
0.13	14,043.16	6,619.99	68.0
0.07	12,795.09	4,091.87	75.8

**Figure 3:**
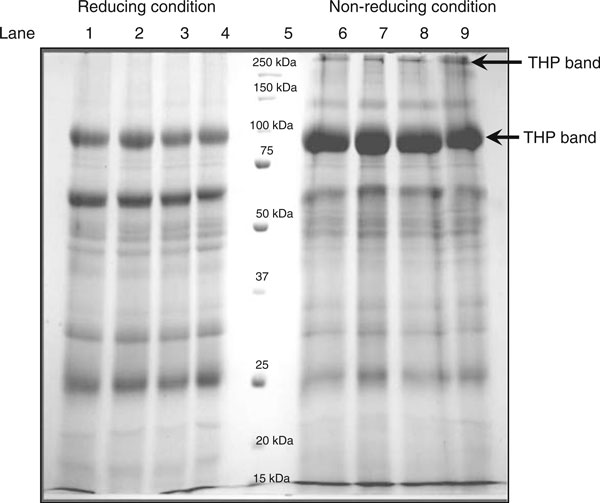
**Urinary proteins profile under reducing and non-reducing conditions**. Lane 1–4: urinary proteins under reducing condition; lane 5: molecular weight markers; and lane 6–9: urinary proteins under non-reducing condition. The gel was Coomassie blue stained. The diagram shows the consistent urinary proteins profiles under reducing and non-reducing condition. The figure shows the SDS-PAGE profile of urinary proteins under reducing and non-reducing conditions.

Aggregation of THP is the major obstacle for analysis of THP using SDS-PAGE. As the result of THP aggregation, they form a large molecular entity that cannot penetrate the resolving gel of SDS-PAGE (Figure 3). In this study, the salting out pellet that contains THP was resolubilised in TSE buffer. TSE buffer was found to be the best resolubilizing buffer to prevent THP aggregation and therefore THP remains in the soluble form. Greater than 90% of THP was found solubilized in TSE buffer (Table 2). Distilled water is a very poor medium to solubilized THP (<10%) whereas <20% of THP solubilized in 0.01 M phosphate buffer saline (PBS) (Figure 2, Table 2).

**Table 2:** Percentage of THP in pellet and supernatant fractions of distilled water, 0.01 M PBS and TSE buffer

	**Intensity (intensity × mm**^**2**^**)**		Percentage of THP (%)	
		
Solubilizing buffer	Pellet fractions (P)	Supernatant fractions (S)	Pellet fractions (P)	Supernatant fractions (S)
Distilled water	1,589.47	88.63	94.7	5.3
0.01 M PBS	3,754.06	760.66	83.2	16.8
TSE buffer	373.92	3,523.15	9.6	90.4

The SDS-PAGE profiles of urinary proteins under reducing and non-reducing conditions are shown in Figure 3. In this study, THP was reduced in 5% β-mercaptoethanol followed by heating at 100°C for 10 min. THP migrated as 97 kDa and 89 kDa proteins under reduced and non-reduced forms, respectively. Reducing of THP was found better for analysis by SDS-PAGE as the reduced form minimizes THP aggregation (Figure 3).

**Figure 4:**
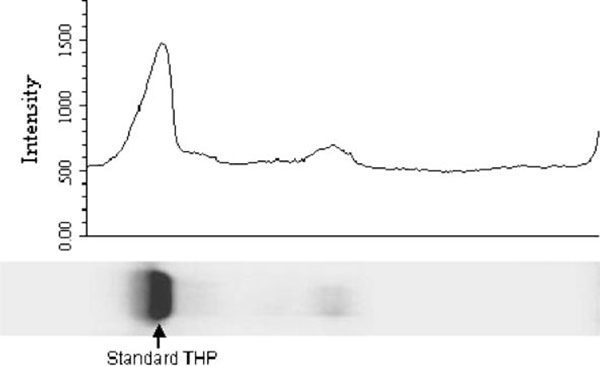
**Densitometry chromatogram of Standard THP**. The intensity of standard THP was 3,253.112.

**Figure 5:**
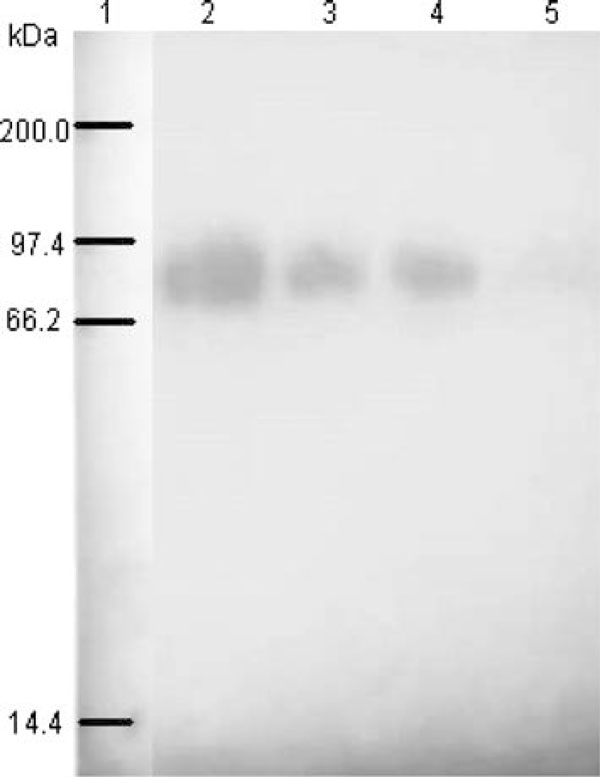
**A single THP band was found at 95 kDa in the immunoblot**. Lane 1: standard THP; lanes 3 and 4: THP isolated from 2 different healthy subjects; and lane 5: THP isolated from a stone former.

**Figure 6:**
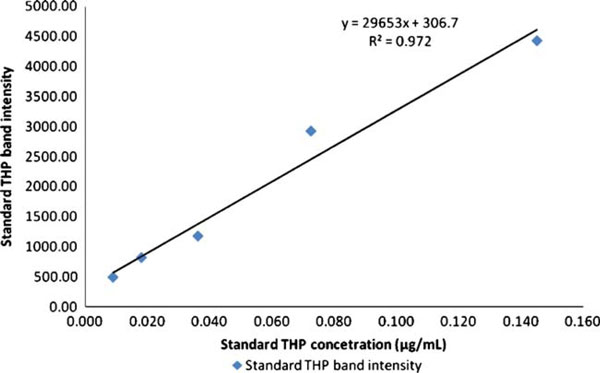
**Standard curve of standard THP in serial dilution ranging 0.018, 0.036, 0.073, and 0.015 μg/mL**.

**Figure 7:**
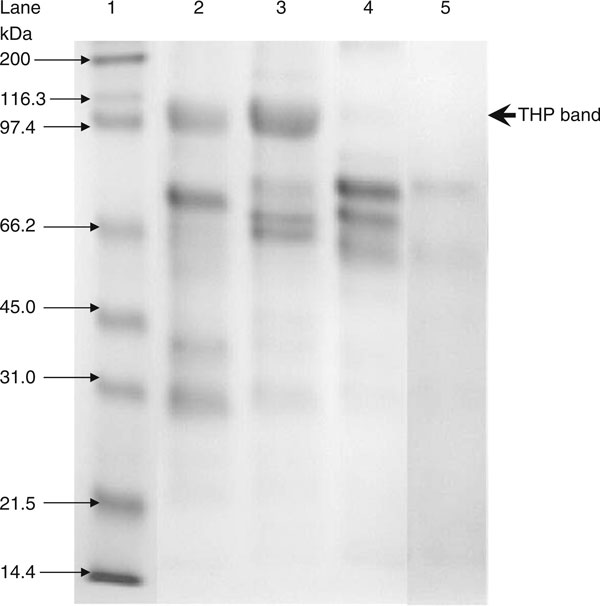
**SDS-PAGE urinary protein profile of standard THP, healthy subject, stone patients**. Lane 1: protein markers; lane 2: healthy subject; lane 3: standard THP; lane 4 and 5: stone patients.

The best loading volume was at 10 μL, where at this volume the THP band's intensity for average healthy subjects was below saturation when compared to the THP standard. This is important as the relative band intensity was the basis of this quantitative method. The electrophoretic migration of standard THP is identical to that of THP isolated from urine. Figure 4 show the densitometry analysis of THP standard while Figure 5 shows the immunoblot result of standard THP and THP isolated from urine. The developed SDS-PAGE method was validated for assay precisions, i.e., reproducibility and repeatability. The assay precisions were 4.8% CV for reproducibility and 2.6% CV for repeatability (Table 3). Figure 6 shows a standard curve for a serial dilution of THP standard, where the densitometry reading of the serial diluted THP bands rise in proportional to the increase in THP concentration. Figure 7 shows an example of SDS-PAGE urinary protein profile in healthy subjects and stone patients. An intense THP band was detected in healthy subject but a very faint band, if detectable, was observed in stone patients.

**Table 3:** Reproducibility and repeatability of SDS-PAGE

Reproducibility (between day assay)	
**OD reading (*n* = 2)**	**Mean THP concentration of QC (μg/mL)**

Day 1	0.29
Day 2	0.27
Day 3	0.27
Day 4	0.27
Day 5	0.26
Average	0.27
Standard deviation (SD)	0.01
Coefficient of variation (CV)	4.8%

**Repeatability (within day assay)**	

**OD reading (*n* = 2)**	**Mean THP concentration of QC (μg/mL)**

1	0.30
2	0.28
3	0.29
Average	0.29
Standard deviation (SD)	0.01
Coefficient of variation (CV)	2.6%

The measurement of QC was carried out over 5 days.

**Figure 8:**
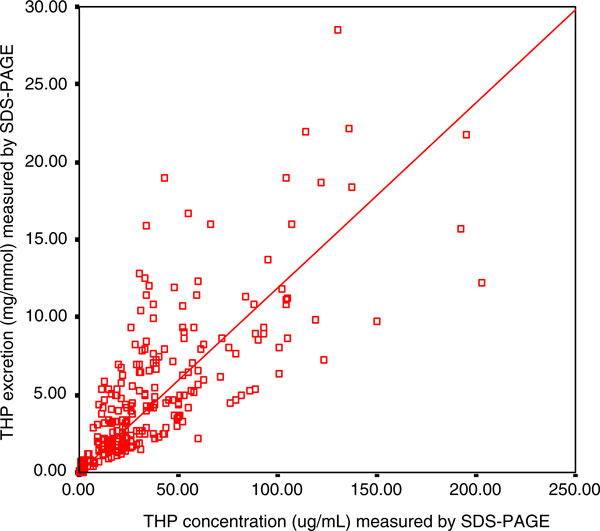
**The correlation between THP concentration and THP excretion measured by SDS-PAGE**. The □ symbols represent the THP level of an individual urine sample with its corresponding THP concentration and THP excretion. The THP concentration was correlated positively to THP excretion (*r* = 0.781, *P* = 0.002). The level of significance according to Pearson correlation of coefficient was set to alpha = 0.01.

**Figure 9:**
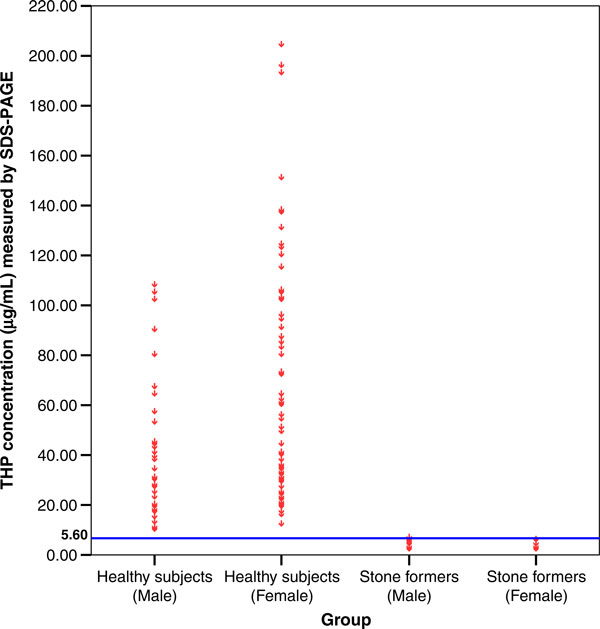
**Establishing of cut-off value for SDS-PAGE**. The о symbols represent an individual urine sample at its corresponding THP concentration. The *horizontal line* was the selected cut-off for SDS-PAGE. The cut-off of THP concentration was set at 5.60 μg/mL. There were 117 healthy subjects and 58 stone patients assayed by this method.

By using the developed SDS-PAGE method, urine samples from 117 healthy subjects and 58 stone patients were analyzed. Figure 8 shows a strong positive correlation (*r* = 0.781, *P* = 0.002) between THP concentrations and THP excretions (THP/Creatinine ratio) as measured by SDS-PAGE. The 95% confident interval of THP concentration in the healthy subjects was 29.34–45.98 μg/mL and 51.86–72.69 μg/mL for male and female, respectively. The THP concentration and THP excretion for female healthy subjects was significantly higher (THP concentration = 62.28 ± 5.23 μg/mL, *P* = 0.0002; THP excretion = 8.89 ± 0.63 mg/mmol, *P* = 0.0004) than that of their counterpart (Table 4). On the other hand, stone patients excreted significantly lower THP concentration (1.71 ± 0.19 μg/mL, *P* = 0.0001) and THP excretion (0.29 ± 0.04 mg/mmol, *P* = 0.0003) than those of the healthy subjects (Table 4) although they did not show any differences in THP concentration in regards to patient gender. The distribution of individual THP concentration of 117 healthy subjects and 58 stone formers is shown in Figure 9. The best fit line to distinguish healthy subjects from stone patients was found at 5.60 μg/mL THP concentration (*P* < 0.05).

**Table 4:** The average age, Creatinine concentration, THP concentration and THP/Creat ratio in healthy subjects, stone patients

	No. of sample	Age (age range) (years)	Creatinine concentration (mmol/L)	THP concentration (μg/mL)	THP/Creat ratio (mg/mmol)
Healthy subjects	117	32 (18–86)	8.14 ± 0.40	53.65 ± 3.84	7.62 ± 0.50
Male	41	52 (19–86)	9.15 ± 0.74	37.66 ± 4.12	5.25 ± 0.68
Female	76	21 (18–60)	7.59 ± 0.46	62.28 ± 5.23^a^	8.89 ± 0.63^b^
Stone patients	58	52 (28–74)	7.94 ± 0.54	1.71 ± 0.19^c^	0.29 ± 0.04^d^
Male	34	52 (36–74)	7.50 ± 0.67	2.03 ± 0.26	0.35 ± 0.05
Female	24	52 (28–68)	8.57 ± 0.88	1.25 ± 0.24	0.21 ± 0.06

All the value expressed as mean ± SE.

^a^Significant compared with male healthy subjects, *P* = 0.0004

^b^Significant compared with male healthy subjects, *P* = 0.0002

^c^Significant compared with healthy subjects, *P* = 0.0001

^d^Significant compared with healthy subjects, *P* = 0.0003

A blind test was conducted on 68 subjects comprising of 26 stone formers and 42 healthy subjects. By using 5.60 μg/mL of THP as the cut-off value to differentiate healthy subjects from stone formers, we found that 2 out of 26 stone formers fall under the false negative category while 7 out of 42 healthy subjects fall under the false positive category. Thus, the sensitivity of the assay was 92.3% while its specificity was 83.3%.

## 4. Discussion

The insoluble substances presence in urine will interfere with electrophoretic migration of proteins in SDS-PAGE and thus hinder accurate analysis of urinary protein pattern [27]. Isolation of urinary protein can be done using ammonium sulphate precipitation as most of the proteins can be precipitated in the presence of high salt concentration [27] leaving behind potential contaminants (e.g., nucleic acids) that cannot be precipitated in urine solution [27,28]. Trace quantity of THP can be effectively recovered using this technique [29]. Ammonium sulphate precipitation is a rapid and cost effective technique for urinary proteins isolation when a large number of urine samples are involved.

Proteins separation in SDS-PAGE is frequently hampered by their self-aggregation. It was documented that THP exists in aggregated state in urine [30]. THP aggregation was influenced by pH, ionic strength, and certain types of cation particularly bivalent cations in its solubilising matrix [31]. TSE is the best buffer to preserve urinary THP in soluble form or even cause dissociation of THP aggregates. The TSE consists of 10 mM Tris, which is a low salt concentration buffer used to prevent THP aggregation; 1% SDS, which is an effective solubilisation agent for THP by disrupting the hydrophobic interactions between THP molecules [28,32]; 1 mM EDTA, which is a protease inhibitor to reduce proteolysis and prevent divalent metal ions chelation and ; pH 8.8, in which THP was preserved in soluble form [7,33]. The composition of TSE buffer has synergistically effect on THP disaggregation and maintaining it in a soluble form.

Under non-reducing condition, the THP molecules appeared in partially aggregated and partially soluble forms. In SDS-PAGE, the non-reduced THP migrated at a lower molecular weight than the reduced THP. The decrease in molecular weight observed is due to a high degree of polypeptide constraint imposed by a large number of intrachain disulfide bridges formed in the non-reduced THP molecule [34]. Reducing of urinary proteins in 1% SDS and 5% β-mercaptoethanol yielded consistent and reproducible protein migration pattern in SDS-PAGE, which serve as one of the criteria for total urinary THP quantification in the present study.

Amount of protein sample loaded onto SDS-PAGE is one of the critical criteria to producing good protein resolution. Since the electrophoretic pattern of protein separated by SDS-PAGE will be affected by the amount of protein loaded, it is important not to overload the gel to avoid a distorted band pattern. In slab gel, overloading of protein in one lane can distort the band pattern in the adjacent lane. On the other hand, an underloaded gel will reduce the detection of trace proteins in the mixture [35]. Hence, the quantity of the protein sample loaded will determine a protein band's sharpness and intensity. By using the stated protein isolation procedure, we found that the optimal sample volume for THP analysis is 10 μL, the gels were stained with Coomassie blue and the color intensity for THP bands is set below the saturation pixel of the imager. By using this approach, we can detect the differences in THP protein concentrations within the sensitivity of Coomassie blue stain [36]. The intensity of the THP band relied on the interaction of the protein with aminotriarylmethane dye of Coomassie Brilliant blue; as dye component may vary from batch to batch, standard THP was run concurrently with the samples in each analysis. The robustness of the assay is proven by the good reproducibility and repeatability of the assay, which were 4.8% and 2.6% CV, respectively.

The method developed was tested on a group of healthy subjects and kidney stone patients. In both the cohorts, urinary THP appeared as a single band and migrated at identical molecular weight on SDS-PAGE. The role of sialic acid content of THP molecule in stone formation has been addressed previously [2,10,37]. Glycosylation of THP in healthy subjects was said to have a protective function on tubular epithelium whilst stone patients excreted defective urinary THP containing less sialic acid and thus diminishing its inhibitory effect on crystal aggregation [10,38,39]. Since sialic acid content on a glycoprotein will interfere with the migration of the glycoprotein in SDS-PAGE, one can predict that the migration of THP isolated from the healthy subjects will be different from those of the stone patients. In this study, we observed a homogeneous migration of urinary THP on SDS-PAGE in both the cohorts, which is an added advantage for the quantification of THP. Hess et al. [37], however, suggested that not only defective THP but a decreased THP excretion may promote the formation of kidney stones.

Urinary creatinine concentration was used as the correction factor for the effect of urine hydration on THP concentration [40]. We used urinary creatinine concentration to estimate THP excretion, which was expressed as THP/Creat ratio. In this study, we found that urinary THP excretion in stone patients was significant lower than that of the healthy subjects. Our finding is in agreement with Yokomizoi et al. [11] although contradictory to Pourmand et al. [20] who reported that no significant difference was detected between the two cohorts. Nevertheless, other studies using radioimmunoassay or ELISA have shown that excretion of THP in stone patients was lower as compared to that of healthy subjects [17-19].

We have shown that the currently developed SDS-PAGE method can be used as a reliable quantitative method for determination of urinary THP concentration. Upon testing with 117 healthy subjects and 58 stone former, 5.6 μg/mL THP concentration (*P* < 0.05) was set as the cut-off point to distinguish stone patients from the healthy subjects. It was then blind-tested on a group of 68 subjects, from which the sensitivity and specificity of the development method was determined to be 92.3% and 83.3%, respectively. Nevertheless, the specificity of the method may be increased if it is used in the context for screening of kidney stone disease when the clinical symptoms for the disease are shown in the patients. Furthermore, this method is simple to perform, cost effective and with high reproducibility. This non-invasive SDS-PAGE-based quantification method may be the answer to diagnosis of kidney stone disease.
